# Neural circuits regulating visceral pain

**DOI:** 10.1038/s42003-024-06148-y

**Published:** 2024-04-13

**Authors:** Xiaoli Chang, Haiyan Zhang, Shaozong Chen

**Affiliations:** 1 College of Acupuncture and Massage, Shandong University of Traditional Chinese Medicine, Jinan, 250355 China; 2 Research Institute of Acupuncture and Moxibustion, Shandong University of Traditional Chinese Medicine, Jinan, 250355 China

**Keywords:** Neural circuits, Gastroenterology

## Abstract

Visceral hypersensitivity, a common clinical manifestation of irritable bowel syndrome, may contribute to the development of chronic visceral pain, which is a major challenge for both patients and health providers. Neural circuits in the brain encode, store, and transfer pain information across brain regions. In this review, we focus on the anterior cingulate cortex and paraventricular nucleus of the hypothalamus to highlight the progress in identifying the neural circuits involved in visceral pain. We also discuss several neural circuit mechanisms and emphasize the importance of cross-species, multiangle approaches and the identification of specific neurons in determining the neural circuits that control visceral pain.

## Abstract (web summary)

In this review, the authors describe the neural circuits that regulate visceral pain to deepen our understanding of visceral pain.

## Introduction

Irritable bowel syndrome (IBS) is the most prevalent disorder of brain–gut interactions and manifests with symptoms such as abdominal pain and a change in stool form or frequency^1,2^. The pathophysiology of visceral pain is incompletely understood, but it is well established that it involves disordered communication between the gut and the brain, leading to motility disturbances, visceral hypersensitivity, and altered central nervous system processing^1,3^. Pain is a complex experience that includes both sensory and emotional aspects^4^. Chronic pain interacts and worsens with the negative emotional reactions it causes. On the one hand, the negative emotional response caused by chronic pain can be used as an indicator of the degree of pain; on the other hand, negative emotions can further aggravate the patient’s pain perception, causing a vicious cycle of pain–negative emotion–pain, which affects the treatment and outcome of pain^5,6^. Interestingly, it has been reported that pain and anxiety processing networks share a subpopulation of lateral septum neurons^7^.

A growing body of research paired with clinical observations supports a critical role of the brain in the generation and maintenance of IBS symptoms. Regardless of the primary symptom triggers, the brain is ultimately responsible for constructing and generating conscious perceptions of abdominal pain, discomfort, and anxiety based on sensory input from the gut^1,2,8^. Specific brain functions, such as sensory processing and modulation, emotion regulation, and cognition, are the result of dynamic interactions between distributed brain areas operating in large-scale networks^9,10^. In recent years, the use of chemogenetic and optogenetic tools in neuroscience research has allowed continuous and reversible manipulation of neuron populations in combination with behavioral measurements, which provides powerful evidence for determining the causal relationship between specific neuronal activities and related behaviors^11–13^. As a result, studies are beginning to reveal the complex neural circuits involved in visceral pain and establish that the threshold and magnitude of pain can be readily modulated by interactions between memory, attention, and affective brain circuitry^14–16^. As researchers have focused on different research subjects individually, in this composite review, we aimed to synthesize our separate views on the neural circuit control of visceral pain into a cohesive picture.

Visceral pain is triggered by central targeted stimuli (neonatal stress and posttraumatic stress disorder) or peripherally targeted stimuli (infection and inflammation)^17^. In rodent models, neonatal maternal deprivation (NMD) and colonic anaphylaxis evoked by intraperitoneal injection of chicken egg albumin has been employed to investigate the neural circuits related to visceral pain in IBS^14,16,18–20^. The anterior cingulate cortex (ACC) and paraventricular nucleus of the hypothalamus (PVN) are widely connected with the relevant regions involved in ascending pain transmission and the descending pain inhibition modulation system^21–24^, making them key regions for transmitting and processing pain perception and emotions^25,26^. Therefore, we searched PubMed for available information describing issues related to brain circuits in animal models of visceral pain from database establishment to the present. Keywords included “irritable bowel syndrome” or “visceral pain” or “visceral hypersensitivity”, and “neural circuit” or “anterior cingulate cortex” or “paraventricular nucleus of the hypothalamus”. In this brief review, we focus on these two nuclei and review recent findings on the neural circuits regulating visceral pain in rodents. In addition, we discuss several neural circuit mechanisms that are believed to be the basis for affecting neuronal activities. We further explore the experience of visceral pain in animal studies and propose suggestions for future directions in the field of visceral pain research.

## ACC

**Fig. 1:**
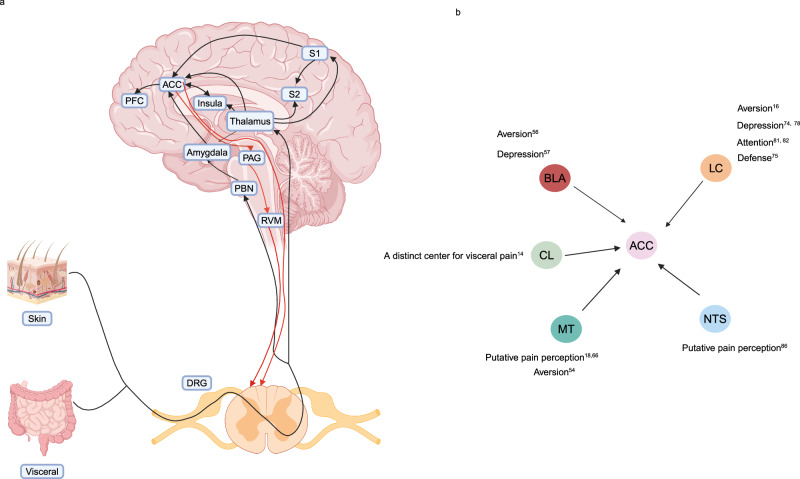
Top-down corticospinal pain and potential neural circuits underlying visceral pain associated with the ACC. **a** Afferent sensory neurons carry information toward the dorsal root ganglion followed by the somatic and visceral nuclei in the dorsal horn. These sensory neurons project directly to the thalamus but also indirectly to the ACC via the PBN and amygdala. Conversely, the ACC can modulate spinal dorsal horn neurons both directly and indirectly via the PAG and RVM. In addition, the ACC also has extensive connections with other nuclei and is involved in regulating pain-related sensory and emotional information. The black arrows indicate ascending projections from the dorsal horn to higher brain structures. The red arrows indicate the indirect and direct descending pathways from the ACC to the dorsal horn. **b** The input brain regions connected to the ACC regulate visceral pain in rodents. The ACC receives inputs from BLA, MT, CL, LC, and NTS neurons and integrates pain perception, aversion, depression, attention, and other information to control visceral pain. Each oval represents a brain area. DRG: dorsal root ganglion; PBN parabrachial nucleus; PAG: periaqueductal gray; ACC: anterior cingulate cortex; RVM rostral ventromedial medulla; PFC prefrontal cortex; S1 somatosensory cortex 1; S2 somatosensory cortex 2; BLA basolateral nucleus of the amygdala; MT medial thalamus, CL claustrum; LC: locus coeruleus; NTS nucleus tractus solitarius (The illustration was created by Xiaoli Chang using BioRender.com under a publishing license).

The ACC is a core brain region that processes the sensory and emotional components of chronic pain in rodents and humans^27,28^. Functional magnetic resonance imaging studies have revealed that the ACC is activated in patients with IBS^29,30^. Similarly, animal studies have shown an enhanced response of the ACC to colorectal distension in visceral hypersensitive rats^31^. Further studies have shown that glutamatergic neurons in the ACC are directly involved in the regulation of visceral sensitivity^32,33^. The connectivity between the ACC and other brain regions as well as descending projections to the spinal cord establish a top–down corticospinal network involve in pain modulation and the integration of emotion with painful experiences^21,34^. Afferent sensory neurons carry information toward the dorsal root ganglion and the somatic and visceral nuclei in the dorsal horn. These sensory neurons project directly to the thalamus and indirectly to the ACC via the parabrachial nucleus (PBN) and amygdala^35–37^. Conversely, the ACC can modulate spinal dorsal horn neurons both directly and indirectly via the periaqueductal gray (PAG) and rostral ventromedial medulla (RVM)^22,38,39^. The ACC has extensive fiber connections with multiple brain regions, such as the amygdala, nucleus tractus solitarius (NTS), insular cortex, locus coeruleus (LC), substantia nigra, and hippocampus^38,40–42^. These complex neural circuit connections provide a fine anatomical basis for the ACC to regulate visceral hypersensitivity in patients with IBS (Fig. 1).

### The basolateral nucleus of the amygdala (BLA) projecting to ACC neurons regulates visceral pain

The amygdala has been shown to exhibit altered activation in response to visceral stimulation in patients with IBS^43^. The BLA, the largest nucleus in the amygdala, is involved in receiving, integrating and encoding pain information and is the center of pain signal processing between the limbic system and the cortical area^44–46^. Many animal experiments have shown that the synaptic activity of BLA glutamatergic neurons is increased in IBS rats and mice^47,48^.

The anterior part of the BLA has dense, direct and reciprocal connections with areas of the ACC^49,50^. The induction of synaptic plasticity is favored by the coordinated timing of action potentials across populations of neurons with rising oscillations of different frequencies, recorded as local field potentials (LFPs)^51^. Considerable evidence suggests that theta rhythms (4-10 Hz) are involved in facilitating the transfer of information between brain regions and in pain perception^52,53^. Furthermore, human studies have shown that experimentally induced acute and chronic pain perceptions can be influenced by transcranial magnetic theta burst stimulation^54,55^. Multiple-electrode array recordings indicated a reduction in long-term potentiation (LTP) in BLA-ACC synapses and an impaired phase relationship between ACC spikes and BLA theta oscillations in rats with visceral hypersensitivity evoked by intraperitoneal injection of chicken egg albumin^19^. Cross-correlation analysis revealed that visceral hypersensitivity led to suppressed synchronization of theta oscillations between the BLA and ACC, suggesting that these regions loosely interact in dynamic information transfer, which may in turn disrupt neural network assemblies and affect synaptic plasticity^19^.

Notably, although these electrophysiological findings were obtained in rat models with visceral hypersensitivity, they were not directly related to visceral pain behavior, which was a potentially relevant confounding factor. Directly assessing visceral pain-related electrophysiological changes in animals will provide useful evidence in future studies. In addition, inhibition of BLA-ACC inputs elicited aversion in animals with neuropathic pain, while activation of BLA-ACC inputs alleviated this aversion^56^. The latest literature has shown that neuropathic pain-induced depression triggers hyperactivity in BLA neurons projecting to the ACC and increases functional connectivity between the ACC and BLA, which was necessary for the selective expression of chronic neuropathic pain-induced depression-like behavior. Moreover, repeated activation of the BLA-ACC pathway was sufficient to activate the main neuronal cell types in the ACC and caused animals to gradually develop depression-like phenotypes^57^. However, in this model, the BLA-ACC pathway was not necessary for mechanical hypersensitivity or persistent pain-like behavior. The regulation of pain by the ACC may depend on brain regions other than the BLA^57^. We hypothesize that the BLA-ACC pathway may regulate emotional experiences that aggravate visceral pain. However, whether the BLA-ACC pathway is a neural circuit that is specific to or necessary for visceral pain requires additional research.

### Claustrum (CL) projection to ACC neurons regulates visceral pain

The CL, an enigmatic brain structure between the insular cortex and the striatum, is reciprocally connected with almost all cortical areas, including the motor, somatosensory, and prefrontal cortices^58,59^. CL neuronal activation strongly contributed to the discrimination of stressed brains after exposure to acute stressors. Chemogenetic activation of stress-responsive CL excitatory neurons mediates anxiety-related behaviors, whereas silencing of the stress-responsive CL neuronal ensemble partly protects against stress-related behaviors^58^. Moreover, colorectal distension stimulation induced a dramatic increase in c-Fos expression in the CL and in synaptic transmission in NMD mice, a well-established mouse model of visceral hypersensitivity^14^. Optogenetic enhancement of CL activity produced visceral pain sensitization, while optogenetic suppression of CL activity attenuated visceral pain sensitization. CFA injection did not alter the excitability or synaptic transmission of CL neurons in mice with CFA-induced somatic pain^14^. These findings suggest that the CL may be a specific nucleus for regulating visceral pain.

There is a neural circuit relationship between the CL and ACC^49,60^. Using an integrative approach of viral tracing, optogenetics, chemogenetics and electrophysiology, Xu et al. evaluated the role of the CL-ACC neural circuit in visceral pain behavior^14^. The results showed that inhibition of CL glutamatergic activity inhibited ACC neural activity and visceral hypersensitivity in NMD mice, while activation of CL glutamatergic activity enhanced ACC neural activity and induced visceral pain in control mice. However, optogenetic regulation of CL-ACC glutamatergic neurons did not alter the inflammatory pain response in mice^14^. Although the ACC receives both somatic and visceral sensory information, the CL may receive visceral pain input, which helps the ACC perceive different information. Determining whether CL GABAergic neurons are activated by colorectal distension stimulation and how the CL identifies visceral pain and somatic pain are key research directions for the future.

### Medial thalamus (MT) projection to ACC neurons regulates visceral pain

Microstimulation of the thalamus can evoke visceral pain, such as angina or labor pain, sometimes years after the original episode^61^. The MT serves as a major relay in the medial pain system and in the conveyance of nociceptive information to the ACC. The MT-ACC circuit has been demonstrated to be involved in pain processing, particularly affective pain perception^62–64^. Meda et al. reported that activation of the MT-ACC exacerbates neuropathic pain-related aversion but has no effect on animals without pain^56^. Recently, increasing research has shown that activation of the MT and ACC is related to visceral pain in IBS^18,65^.

IBS can affect the activation of the MT and ACC and induce corresponding synaptic signal transmission. Electrophysiological recording revealed long-lasting potentiation of the LFPs at MT-ACC synapses in rats with visceral hypersensitivity evoked by intraperitoneal injection of chicken egg albumin^18^ or repetitive distension of the colon and rectum^66^. Theta burst stimulation in the MT reliably induced LTP in the MT-ACC pathway in normal rats but was occluded in rats with visceral hypersensitivity. Furthermore, repeated tetanization of the MT increased ACC neuronal activity and visceral pain responses in normal rats, mimicking those of rats with visceral hypersensitivity^18,66^. Cross-correlation analysis revealed that augmented synchronization of the MT-ACC theta-band LFPs was consistent with increased neuronal communication between the two regions^66^. However, these findings lack direct links to visceral pain perception and emotional behavior.

In addition, acupuncture, a promising nonpharmacological treatment, significantly improved the pain threshold induced by rectal expansion and improved the related clinical symptoms of IBS^67,68^. Huangan Wu’s team observed that electroacupuncture at the Tianshu and Shangjuxu acupoints alleviated visceral hypersensitivity in rats with IBS induced by neonatal colorectal stimulation through the inhibition of astrocyte activation in the MT and ACC^69^. However, whether electroacupuncture improves visceral pain by regulating the activity of astrocytes in the MT-ACC pathway has not been determined. Based on the above results, we speculate that the MT-ACC pathway is involved in the regulation of visceral pain, but many questions still need additional detailed research.

### LC projection to ACC neurons modulates visceral pain

The LC noradrenergic system is the main source of noradrenaline in the central nervous system and is involved intensively in modulating pain, attention, arousal, learning and memory^70–72^. Acute pain triggers a robust LC stress response, producing spinal cord-mediated endogenous analgesia while promoting aversion, vigilance, and threat detection through ascending efferents^71^. Activation of LC-spinal cord noradrenergic neurons reduced hind-limb sensitization and induces conditioned place preference^72^. In contrast, activation of LC-prefrontal cortex noradrenergic neurons exacerbated spontaneous pain, produced aversion and exacerbates anxiety-like behavior^72,73^. These results have shown that the regulation of pain in different neural regions is not homogeneous.

The ACC is an important brain region for negative emotional memory storage and receives extensive projections from the LC^74,75^. Aversion and distress can be measured via the two-chamber conditioned place avoidance assay (CPA) or poststimulation ultrasound vocalization recording. Combining colorectal distension with CPA directly reflects the affective component of pain evoked by visceral stimulation and leads to considerable aversion in associative learning and memory^76,77^. Selective ablation of LC noradrenergic neurons disrupted the formation of visceral noxious aversive memories. Further research has shown that optogenetic activation or silencing of LC neurons projecting to the ACC could enhance or reduce learning and memory formation, respectively, as well as the expression of c-Fos in the LC and ACC, respectively^16^. These data indicate that norepinephrine derived from the bottom-up LC-to-ACC neuronal pathway is responsible for inducing aversive behavior. Moreover, alterations in LC-ACC connectivity were negatively correlated with depression severity^78^. The chemogenetic blockade of LC noradrenergic neurons projecting to the ACC completely reversed depressive-like behavior^74^.

Recent studies have demonstrated that LC-ACC noradrenergic neurons facilitate excitatory synaptic transmission to pyramidal cells in the ACC and enhance itch- and pain-like responses in animals^75^. Considering the crucial role of the LC in cognitive processes such as learning and memory and arousal^79,80^, we speculate that ascending projections in the LC-ACC may enhance behavioral responses to injury in animals or humans and alert them to dangerous situations. In addition, the circuit from the LC to the ACC played a crucial role in sustained attention and offspring interactions in mice^81,82^. In conclusion, LC-ACC noradrenergic neurons may improve visceral pain by regulating multiple behaviors, such as aversion, depression, attention and defense.

### Ascending projections from the NTS to the ACC regulate visceral pain

Visceral information is transmitted to the NTS through the bilateral vagus nerves and subsequently relayed to higher brain centers, where it ultimately reaches the limbic system and cognitive brain centers, which are thought to mediate the emotional aspects of pain^83–85^. The caudal two-thirds of the NTS is a major hub for general visceral sensation^86^. Increasing evidence has demonstrated that noxious gastrointestinal and pancreatic stimuli induce the overexpression of c-Fos in the NTS^41,86,87^. Our laboratory observed an abnormally elevated theta oscillation in the NTS of IBS rats during colorectal distension. After colorectal distension ended, the central post-response effect was longer (data not published). In rats with chronic pancreatitis induced by trinitrobenzene sulfonic acid, excitatory synaptic transmission within the NTS was potentiated. Inhibiting both the excitatory synaptic transmission and neural activity of NTS neurons alleviated visceral hypersensitivity in chronic pancreatitis rats^86^.

In chronic pancreatitis rats, some c-Fos-expressing neurons in the NTS projected to the ACC, which provides evidence that the cortical pain center could be directly activated by visceral afferents processed from the NTS. In addition, a larger portion of ascending fibers from the NTS innervated CaMKII-expressing neurons than GAD-67-expressing neurons in the ACC^41,86^. In summary, the NTS-ACC pathway may be an essential component of the ascending system involved in the transmission of abdominal hyperalgesia in chronic pancreatitis rats. However, whether this neural circuit is necessary for regulating visceral pain needs further study.

### Possible mechanisms

#### N-methyl-D-aspartate receptor (NMDAR) and plasticity

NMDAR is a major type of ionotropic glutamate receptor in the central nervous system that plays a fundamental role in both synaptic transmission and plasticity^88^. In the ACC, NMDAR-dependent LTP plays an important role in injury-triggered cortical excitation and plastic changes. There were significantly more NMDARs, especially the NR2B subtype, and CaMKIIa, a protein kinase critical for neuronal plasticity that is downstream of NMDAR, in the ACC postsynaptic density region in rodents with visceral hypersensitivity than in control rodents^14,41,89^. NMDARs mediate ACC synaptic responses after the induction of visceral hypersensitivity. The NMDAR antagonist AP5 or the CaMKII inhibitor KN-93 markedly inhibited ACC neuronal firing evoked by 50 mmHg colorectal distension and visceral pain responses in NMD mice^14^. When an external stimulus or experience activates an NMDAR, it can cause an influx of extracellular Ca^2+^ through the NMDAR, triggering a cascade of signaling molecules, including CaMKIIa, protein phosphatases, and enzymes that produce diffusible retrograde messengers^90^. Phosphorylated CaMKII binds to and stabilizes postsynaptic NR2B, mediating visceral pain in rats with visceral hypersensitivity induced by intraperitoneal injection of chicken egg albumin^91^. Further studies have confirmed that NMDARs are involved in the regulation of the CL-to-ACC circuit in visceral pain^14^. Taken together, these data suggest that excessive NMDAR subunits and overactive CaMKII in the ACC region are involved not only in the processes of modifying visceral pain and aversive responses to pain but also in the further processing of learning and memory in the chronic pain state.

#### Myelination and plasticity

Myelination accelerates nerve impulse propagation along the axon, facilitating long-range oscillations and synchronous spike-time arrivals between neurons in different brain regions and promoting plasticity^51,92^. Recent evidence suggested that myelin formation was impaired in human subjects and animal models of chronic visceral pain^93,94^. Repeated activation of the BLA-ACC pathway impaired myelination and triggered depressive-like behaviors in naive mice^57^. After demyelination, the differentiation of oligodendrocyte progenitor cells contributes to effective myelin regeneration, while mature oligodendrocytes may cause inefficient and mistargeted myelin regeneration to a lesser extent^95^. Chronic visceral hypersensitivity downregulated the MyRF gene, which encodes a transcription factor necessary for the differentiation of immature oligodendrocytes to mature oligodendrocytes, preventing oligodendrocyte maturation and myelination and ultimately resulting in the hypomyelination of the ACC in rats with visceral hypersensitivity evoked by intraperitoneal injection of chicken egg albumin^94^. Astrocytes promote myelin structure and conduction velocity by directly participating in the proliferation, differentiation and migration of oligodendrocytes^93^. Previous studies have shown that the development of reactive astrogliosis in rats with chronic visceral hypersensitivity is induced by the intraperitoneal injection of chicken egg albumin^94,96^. Astrocytic Gq pathway activation in the ACC induced oligodendrocyte progenitor cell proliferation and differentiation and enhanced oligodendrocyte regeneration, maturation, and myelination, which repaired visceral hypersensitivity-induced impairments in ACC–amygdala spike-field synchrony^94^. However, how these functions are related to specific oligodendrocyte states and how they are influenced by internal and/or external factors (such as peripheral inflammation, the gut microbiota and motility) are unclear.

#### Astrocytes and plasticity

Astrocytes are considered key cells that combine synaptic activity with energy metabolism and are the main contributors to aerobic glycolysis in the brain. Damage to aerobic glycolysis in astrocytes can lead to impaired synaptic plasticity^97–99^. Astrocytes have fine synaptic-perisynaptic processes that encapsulate synapses in a dynamic manner and form a “tripartite synapse” structure with presynaptic and postsynaptic neurons^100,101^. Adjacent astrocytes communicate through gap junction channels, delivering energy-supplying substances or neurotransmitters such as lactate to other astrocytes to exert a wider range of effects, effectively creating a distributed energy pool for sharing and delivering energy to active synapses as needed. The ideal location of astrocytes enables them to perceive weak changes in the surrounding environment in response to transiently elevated energy requirements associated with neuronal activation^102,103^.

Activation of astrocytes in the ACC elicits lactate release, improves decision-making in the physiological state, and rescues impaired decision-making and aversive memory formation and consolidation in rats with chronic visceral pain^96^. The activation of astrocytes rescues ACC synaptic LTP and repairs impaired spike phase locking in rats with chronic visceral pain^96,104^. Synaptic plasticity and synchronized brain network activity require additional energy support. Lactate released by astrocytes is used as an energy supply for synaptic activity in neurons to produce and store memory^96,105^. In addition, lactate can also act as a signaling molecule to affect the expression of synaptic plasticity-related proteins, including pCREB, Erk, c-Fos, Zif268 and BDNF, in cortical neurons^104,106^. Activation of the norepinephrine β2 receptor, which is widely expressed in astrocytes, promoted glucose uptake and may regulate astrocyte glucose metabolism, such as lactate production or transduction^107^. In another study, knocking down astrocytic but not neuronal β2ARs in the ACC impaired CPA memory, indicating that β2ARs expressed by ACC astrocytes rather than neurons may be critical effectors of adrenergic-mediated effects on aversive memory formation^16^. In addition, astrocytes can modulate synaptic activity by the release of gliotransmitters such as glutamate, GABA, adenosine-5’-triphosphate and D-serine, which bind to an array of pre- and post-synaptic neuronal receptors and influence synaptic transmission and plasticity^108,109^. To learn more about how astrocytes can affect synaptic activity and plasticity through gliotransmission, the reader is referred to some excellent reviews^109,110^. Gliotransmission was enhanced in reactive astrocytes in chronic pain^111^. The role of glial transmission in chronic visceral pain in IBS needs further evidence. In conclusion, these data indicate that ACC astrocytes can produce specific effects on surrounding neurons by modulating neuronal activity and that astrocytic signaling in the ACC network is necessary for promoting synaptic plasticity and network communication.

## PVN

**Fig. 2:**
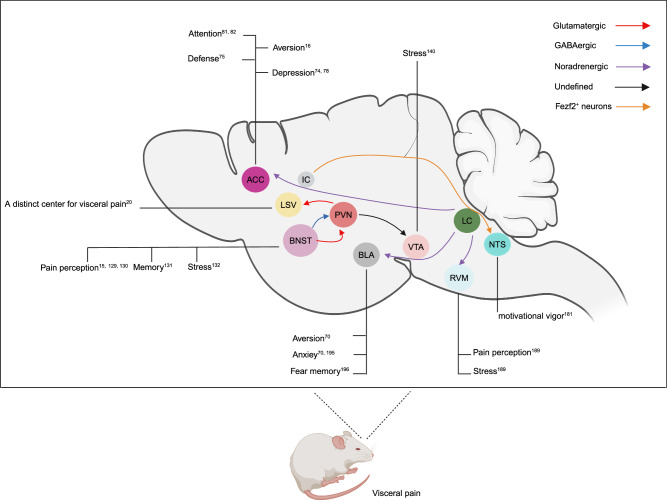
Typical neural circuits involved in visceral pain in rodents. The PVN receives glutamatergic and GABAergic inputs from the BNST; provides inputs to the LSV and VTA; and integrates pain perception, stress and memory to regulate visceral pain. LC noradrenergic neurons can form complex neural circuits by projecting to multiple brain regions, such as the RVM, BLA, and ACC, to jointly regulate pain perception and emotion and thus play an important role in visceral pain. Insular cortex neurons expressing Fezf2 selectively control motivational vigor and invigorate need-seeking behavior through projections to the brainstem NTS, which may also be involved in the regulation of visceral pain. Each oval represents a brain region, and different line colors represent different projection types. PVN Paraventricular nucleus of the hypothalamus, BNST Bed nucleus of the stria terminalis, LSV Ventral of lateral septal, VTA Ventral tegmental area, LC Locus coeruleus, RVM Rostral ventromedial medulla, BLA Basolateral nucleus of the amygdala, ACC anterior cingulate cortex, NTS Nucleus tractus solitarius, IC Insular cortex (The illustration was created by Xiaoli Chang using BioRender.com under a publishing license).

In the central nervous system, the PVN contains a collection of neurosecretory and nonneurosecretory cells. Neuroendocrine cells include parvocellular and magnocellular neuroendocrine cells; the former type of cells are mainly responsible for the synthesis of corticotrophin-releasing hormone (CRH), and the latter type of cells are responsible for the secretion of vasopressin and oxytocin^112,113^. CRH neurons within the PVN play a pivotal role in the pathogenesis of visceral hypersensitivity induced by CRD^26,114^. Hypothalamic CRH neurons are involved in both basal and stress-induced hypothalamic–pituitary–adrenal (HPA) axis activation. Exogenous administration of CRH exacerbates gastrointestinal responses in patients with IBS and rats, whereas CRH receptor antagonists reverse these responses^115,116^. Efferent connections to the spinal dorsal horn directly or indirectly through various relay nuclei in the brainstem, such as the PAG and RVM, provide a potential anatomical substrate for the descending antinociceptive action induced by the PVN^24^. In addition, PVN neurons are strongly connected to multiple nuclei, including the NTS, PBN, amygdala, bed nucleus of stria terminalis (BNST), nucleus accumbens, lateral sulcus, prefrontal cortex (PFC), arcuate nucleus, ventral tegmental area (VTA), and others^15,117–120^, providing an anatomical basis for the important role of the PVN in pain transmission. Recently, Liu et al. proposed that activation of oxytocinergic signaling in the PVN-PFC circuit reduces acute and chronic pain^121^. However, compared to the research on the regulation of visceral pain in PVN CRH neurons, the results of this study are insufficient. Therefore, in this review, we investigated the role of only PVN CRH neurons and their neural projections in visceral pain (Fig. 2).

### PVN input to the ventral of lateral septal (LSV) nucleus controls visceral pain

The lateral septal (LS) is implicated as a hub that regulates affective behaviors, such as feeding, reward, anxiety, fear, cognition and memory^7,20,122^. Recent studies have shown that the LS is also involved in the specific regulation of visceral pain^20^. Noxious stimulation of the viscera caused visceral pain and enhanced regional cerebral blood flow in the LS in rats^123^. Notably, visceral rather than somatic stimulation significantly enhanced c-Fos expression and calcium activity in LSV CaMKIIa-positive neurons in NMD mice. Optosilencing of LSV CaMKIIa-positive neurons attenuated colorectal distention-evoked visceral pain in NMD mice, whereas optoactivation of these neurons promoted colorectal distention-evoked visceral pain behaviors in normal animals^20^.

The LSV receives projections from the PVN and regulates feeding and hypersomnia behaviors^15,124,125^. Further studies have indicated that the PVN exhibits anterograde projections of CaMKIIa-positive neurons to the LSV regions, which are specifically involved in the regulation of visceral pain induced by NMD^20^. Inhibition of the PVN-LSV pathway attenuated colorectal distention-evoked visceral pain responses, whereas activation of the PVN-LSV pathway contributed to adaptive colorectal distention-evoked visceral pain responses^20^. Taken together, these results demonstrate the critical role of CaMKIIa-positive neuronal connections between the PVN and LSV regions in visceral hyperalgesia. Targeting this unique pathway may provide a potentially powerful approach for the clinical treatment of visceral pain in patients with IBS.

### The BNST-PVN circuit regulates visceral pain

The BNST, a key part of the extended amygdala, is a gray matter zone located inside the caudate nucleus that contains many subregions and specific nerve cells. The BNST has a significant effect on mood because it is associated with autonomic and neuroendocrine functions^126^. The BNST regulates physiological responses to stress not only via its own preautonomic projections but also through its direct connections to the PVN^127^. The neural circuit from the BNST to the hypothalamus region can promote eating and drinking behavior and ensure the maintenance of the body’s internal steady-state environment through connections with the brainstem^128^.

Accumulating evidence has confirmed that the PVN receives inhibitory neuronal projections from the BNST, especially the anteroventral BNST (avBNST)^127,129^. In rats with visceral hypersensitivity induced by neonatal colorectal distension or NMD, the excitability of GABA neurons in the avBNST projecting to the PVN was attenuated^129,130^. Furthermore, destruction of GABAergic avBNST-PVN neurons facilitated the activation of CRH neurons in the PVN and the development of visceral pain^129,130^, while activation of GABAergic avBNST-PVN neurons inhibited the activation of CRH neurons in the PVN and alleviated visceral pain^130^. These findings further authenticate the pivotal role of PVN-projecting GABAergic neurons in the avBNST in the modulation of visceral hypersensitivity. There is extensive evidence that glucocorticoid hormones enhance memory consolidation, helping to ensure that emotionally significant events are remembered. avBNST activity alters consolidation via the regulation of glucocorticoid secretion through GABAergic input to the HPA effector region of the PVN^131^. Decreased activity in this pathway may provide an endogenous mechanism for augmenting HPA output and memory consolidation following an emotionally arousing event.

Notably, glutamatergic neurons in the avBNST also input to PVN CRH neurons^15^. Recent results have indicated that the balance between excitatory and inhibitory synaptic inputs is altered in mice susceptible to visceral hypersensitivity, resulting in excitation of PVN CRH neurons. Chemogenetic activation of GABAergic neurons or inhibition of glutamatergic neurons in the avBNST-PVN circuit alleviated visceral hypersensitivity^15^. Interestingly, the study further revealed that inhibition of the ventromedial hypothalamus-PVN/nucleus accumbens-PVN circuit had no effect on visceral hypersensitivity in susceptible mice. Therefore, the unique avBNST-PVN neural circuit helps PVN CRH neurons modulate visceral pain.

Previous work has shown that early traumatic events are associated with lower BNST-PVN connectivity, which may indicate that the BNST has a diminished capacity to constrain the PVN and stressor-evoked HPA responses, perhaps yielding greater stress reactivity^132^. In addition, clinical diffusion tensor imaging data indicated stronger BNST-hypothalamus structural connectivity in women, which may underlie sex differences in symptoms related to abstinence from alcohol and risk of relapse^133^. We hypothesize that the BNST-PVN circuit plays a key role in sex differences in visceral pain in diseases such as IBS, but this requires further research. In brief, the avBNST-PVN circuit may play a crucial role in the regulation of visceral pain by participating in pathways related to memory, stress, and pain perception.

### PVN input to the VTA regulates visceral pain

The VTA is one of the two main regions involved in dopamine synthesis and release in the central nervous system. It plays an important role in cognitive, emotional, learning and memory, reward and defensive behaviors^134–136^. Recent evidence suggests that the VTA is widely involved in the regulation of chronic pathological pain due to stress^137,138^. Chronic stress increased the mRNA and protein expression of tyrosine hydroxylase (TH), a marker of dopamine neurons in the VTA^139^. Colorectal distension stimulation induced dramatic increases in c-Fos expression and dopamine levels in the VTA^140^.

The VTA receives projections from the PVN that regulate prosocial behaviors and active paternal behaviors^15,141,142^. Inhibition of CRH in the PVN blocked TH production in the VTA in rats with repeated colorectal distension, but basal TH enzyme levels were not affected^140^. Dopaminergic neurons in the VTA constitute a major stimulatory pathway in the stress-induced activation of the HPA axis. The VTA was a probable site for synaptic interactions between CRH and dopaminergic neurons^143^. CRH mediates stress responses induced by colorectal distention in the PVN and activates the mesolimbic dopamine system and CRH-induced dopamine release in the VTA, which further evokes the development of visceral pain.

### Possible mechanisms

#### CRH neurons in the PVN and HPA axis

The abnormal bidirectional communication function between the brain and digestive tract is partly caused by the activation of the HPA axis in the neuroendocrine stress system^144^. Studies have shown that various stimuli can converge on the hypothalamus through the central and peripheral nervous systems, leading to the release of CRH from the PVN to the pituitary portal system, where it acts on the anterior pituitary gland. The pituitary gland releases adrenocorticotropin hormone (ACTH) into the systemic circulation, causing an increase in cortisol release. Under normal physiological conditions, an increase in cortisol can cause negative feedback in the HPA axis^145–147^. This negative feedback mechanism acts on the hypothalamus, pituitary, PVN, and limbic system, inhibiting CRH generation, ultimately closing the HPA axis and restoring the body to homeostasis. As an important neuroendocrine peptide, CRH not only can act on the pituitary gland but can also affect the motility and hormone secretion of the gastrointestinal tract, thereby regulating colon motility and improving visceral sensitivity^148,149^.

Previous evidence has shown that the expression of PVN c-Fos, pituitary and plasma CRH, ACTH and cortisol increase in rodents with visceral hypersensitivity, indicating that the HPA axis is activated^114,150–153^. Additionally, stress-induced activation or enhancement of the CRH and HPA axis systems is associated with visceral hypersensitivity, which is an important feature of IBS. Similarly, patients with IBS exhibit greater reactivity to stress than healthy individuals, as manifested by a dysregulated HPA axis response and enhanced visceral perception and gut motility, among other findings^154,155^. Various stress stimuli can change the neural activity of the PVN, cause sustained high expression and release of CRH, and disrupt the communication of circuits such as the BNST-PVN, PVN-LS, and PVN-VTA circuits, thus inducing anxiety, depression and visceral hypersensitivity.

#### CRH neurons in the PVN and neuroinflammation

Mast cells are recruited and degranulate in enteric disease-related visceral hypersensitivity^156,157^. There is a potential correlation between the activation of abundant mast cells and visceral pain^156,158,159^. Mast cells, which act as the “first responders” of the immune system, reside close to neurons in the central nervous system. The hypothalamus is rich in mast cells. A recent study revealed that NMD-induced visceral hypersensitivity and PVN mast cell activation occur in adulthood. Injection of cromolyn into the PVN to stabilize mast cells alleviated visceral hypersensitivity in a dose-dependent manner and reversed the mast cell activation induced by NMD^160^. In addition, mast cell-deficient Kit^W-sh/W-sh^ mice exhibited an increased pain threshold, suggesting that mast cells contribute to the development of visceral hypersensitivity^160^. Stabilization of mast cells by intra-PVN infusion of the mast cell stabilizer cromolyn not only suppressed visceral hypersensitivity induced by NMD and the mast cell degranulator C48/80 and the hyperactivity of CRH neurons but also attenuated the stimulation-induced activation of mast cells and increase in mast cell degranulation products in the PVN^160^. Taken together, these findings suggest that early-life stress in the form of NMD increases mast cell activation in the PVN and subsequently releases inflammatory mediators involved in CRH-related neuronal sensitization and chronic visceral pain in adulthood. In addition, studies have shown that CRH may also stimulate mast cell activation, leading to sensitization of nerve terminals and increased pain signaling^161–163^. However, further studies will be necessary to explore the mechanism of the interaction between PVN mast cells and CRH neurons in visceral hypersensitivity.

Microglia, immune cells of the central nervous system, play an initiating role in chronic visceral pain^114,164–166^. Studies have confirmed that information transmission and communication between neurons and microglia may regulate pathological pain and visceral pain^26,167^. Rats with visceral hypersensitivity exhibited significant increases in Iba-1 (a protein marker of microglial activation) immunofluorescence and protein expression in the PVN, which could be prevented by intra-PVN infusion of minocycline, a nonselective inhibitor of microglia. Moreover, intra-PVN infusion of minocycline prevents colorectal distension-induced pain threshold reduction and CRH protein elevation^114^. Activated microglia can release many proinflammatory factors or chemokines, such as IL-1β, IL-6, and TNF-α, and promote the activation of PVN CRH neurons. Long-lasting activation of PVN CRH neurons may cause changes in neuronal plasticity, resulting in increased reactivity to pain stimulation^26,114^. These observations suggest that the activation of CRH neurons and microglia in the PVN is involved in the occurrence and development of visceral hypersensitivity.

#### Small-conductance Ca^2+^-activated K^+^2 (SK2) channels in PVN neurons

The excitability of neurons is regulated by ion channels, and abnormalities in these channels are important mechanisms of pain^168^. SK2 channels are voltage independent and can regulate the firing frequency of neurons by giving rise to afterhyperpolarization currents (I_AHPs_) carried by K^+^. Inhibition of SK2 channels increases neuronal excitability and enhances excitatory synaptic transmission, as indicated by an increased frequency of miniature excitatory postsynaptic currents and action potentials, leading to hyperexcitability and pain hypersensitivity^169^. Recent research has shown that visceral hypersensitivity is related to the downregulation of SK2 channel proteins as well as the inactivation of SK2 channels in PVN CRH neurons^15,170,171^. Injecting SK2 channel activators into the PVN can reverse visceral hypersensitivity. Further studies have shown that a decrease in the I_AHPs_ of SK2 channels and the restoration of SK2 expression and function rescue the inhibitory current of CRH neurons in the PVN of susceptible mice, which is crucial for the desensitization of PVN CRH neurons^15^. There are also many ion channels, such as Nav1.7, Kv1.1, HCN, and TRPV4, that play essential roles in neuropathic pain^172–174^. However, whether these ion channels are involved in the regulation of visceral pain in PVN CRH neurons still needs further study.

## Other neural circuits involved in visceral pain

### The insular cortex input to the NTS regulates visceral pain

The insular cortex is a highly polar center that integrates visceral autonomous activities and is known as the “visceral brain”. Imaging studies have shown that, compared with controls, patients with IBS exhibit decreased thickness and lower gray matter volume in the insula, which are associated with a longer disease duration and visceral hypersensitivity^175,176^. The insula is continuously activated during the resting state and during colorectal distension stimulation in patients or rodents with IBS^176,177^. In rats with chronic pancreatitis induced by trinitrobenzene sulfonic acid and NMD, increased c-Fos expression and potentiated excitatory synaptic transmission within the insular cortex were observed. Specifically, inhibiting the excitability of insular pyramidal cells reduced both abdominal hyperalgesia and pain-related anxiety^178,179^. These studies have shown that the insular cortex can be targeted for the treatment of visceral pain.

Animal experiments have confirmed that the insular cortex and NTS have bidirectional projections^180–182^. Deng et al. showed that insular cortex neurons expressing Fezf2 selectively control motivational vigor and invigorate need-seeking behavior through projections to the brainstem NTS^181^. The inhibition or activation of neurons in the insular cortex via the ^Fezf2^-NTS pathway potently suppressed or exacerbated, respectively, motivational vigor without influencing food or fluid consumption or affecting valence processing, indicating that the function of this circuit is remarkably specialized^181^. Recent research has shown that the insular cortex stores immune-related information. Chemogenetic reactivation of these neuronal ensembles was sufficient to broadly retrieve the inflammatory state under which these neurons were captured^183^. Furthermore, the insular cortex-NTS neural circuit is an important neural pathway for brain-gut communication. Through this pathway, the projection of the NTS to the insular cortex can transmit the discomfort of the gastrointestinal tract to the central cortex, thus affecting central sensitization; the projection of pyramidal neurons from the insular cortex regulates the activity of neurons in the medullary vagal complex, thereby regulating gastrointestinal function and affecting peripheral pain sensitization. The visceral sensory information and emotional responses transmitted by the NTS converge in the insular cortex, and mutual influence leads to central sensitization to pain regulation in the insular cortex. Oscillation synchronization activity and regulation of the insular cortex-NTS neural circuit have not been reported in visceral pain in IBS animals, which indicates that further research is necessary.

### LC input to the RVM mediates visceral pain

The RVM is part of the descending pain modulation system, which exerts ‘top-down’ control on ascending nociceptive transmission and plays an important role in the initiation and maintenance of neuropathic pain^184,185^. Several studies have shown that the RVM plays a major role in the central processing of visceral pain, such as urinary bladder distention^186^, pancreatic pain^187^, colorectal distention and visceral hyperalgesia induced by chemical intracolonic irritants^188,189^. The number of neurons immunoreactive for the c-Fos protein and NR1 was significantly greater in the RVM in IBS rats than in control rats^190,191^.

Previous studies have shown a functional connection between the RVM and LC. The RVM and LC neurons were altered in response to stress stimulation or visceral noxious stimulation^192,193^. Recent studies have shown that LC noradrenergic neurons send monosynaptic projections to the RVM; these projections are predominantly located in the dorsal part of the LC^189^. Selective activation of the LC-RVM noradrenergic neuron circuit in naive mice instantly induced stress-related psychiatric disorders and subsequent visceral hyperalgesia. In contrast, selective inhibition of LC-RVM noradrenergic neurons attenuated psychological maladaptation and pathological visceral hyperalgesia induced by stress or inflammation without affecting physiological pain^189^. Research has shown that microinjection of α1 agonists into the RVM increased the nociceptive response, while microinjection of α2 agonists reduced the nociceptive response^194^. Therefore, the increased activation of α1-adrenoceptors from the LC on RVM neurons might lead to visceral hypersensitivity in mice with visceral pain. In addition, activation of LC noradrenergic neurons that project to the BLA evoked norepinephrine release in the BLA, altered BLA spike firing and condition aversion^70^, increased anxiety-like behavior^70,195^ and impaired extinction of fear memories^196^. Taken together, these findings suggest that LC-related noradrenergic neurons can form complex neural circuits by projecting to multiple brain regions, such as the RVM, BLA, and ACC, to jointly regulate pain perception and emotion and thus play important roles in visceral pain (Fig. 2).

## Future research directions

### Establishing a reliable and replicable animal model of visceral pain

In comparison to somatic pain, visceral pain can be slightly more difficult to measure and model. The existing animal models have certain drawbacks that limit their application in laboratory research and translational medicine. With the NMD model, there are many variations in separation duration in terms of hours/day and length of protocol across the neonatal period^197,198^. In addition, the development of NMD models can be costly in terms of animal housing for the duration of the study, which is a major consideration for budgetary resources. Therefore, it is necessary to design, summarize and demonstrate additional ideal animal models and further promote research in this field. In addition, there is currently no objectively recognized standard for evaluating visceral pain models. It is also worth noting that when different factors induce visceral pain in animal models, the neural circuits responsible for encoding pain signals may also be inconsistent.

### A multi-sex, cross-species scientific study

Studies have shown that women are 1.5–3.0 times more likely to have IBS than men^1,199^. However, the current experimental results on the neural circuits involved in visceral pain were obtained from male rodents and may not be fully applicable to female rodents. It is possible that female animals were not used in these studies because of the effect of the menstrual cycle and estrogen on visceral pain^200,201^. It is worth exploring whether the neural circuits we reviewed above exhibit sex differences in the regulation of visceral pain in diseases such as IBS. In addition, whether the knowledge acquired in rodents can be extended to humans is a key question. There are many challenges in breaking down the barriers between preclinical animal researchers and clinical investigators studying human subjects. However, cross-species comparisons must be performed to test this hypothesis.

### Identification of specific functional neurons in the neural circuits involved in visceral pain

Usually, different neuronal populations are mixed within the same brain region, but they regulate pain perception and/or emotional aspects of visceral pain by projecting to different downstream targets. Qiufu Ma’s team showed that spinal vesicular glutamate transporter 3 lineage neurons are specifically required to drive affective pain^77^. However, whether there are specific neurons in the central nervous system that mediate somatic pain or visceral pain and whether there are specific neurons that mediate pain perception and emotion will be interesting topics of future research. The use of single-cell RNA sequencing profiling and projection specificity to catalog neuronal cell types will promote the development of this research. As more types of neurons are identified, in the future, we can conduct parallel studies on each type to determine how different neurons integrate internal and external cues to regulate visceral pain while providing more accurate targets for the treatment of visceral pain.

### How complex neural circuits collaborate to control visceral pain

The brain nuclei do not exist in isolation but rather form complex and fine neural networks with each other. Visceral pain is a vast, complicated, and fascinating topic. In addition to the neural circuits involved in visceral pain mentioned in this review, there are many other mysteries about communication between neurons in different brain regions and local microcircuits in nuclei that need to be further elucidated. More importantly, the neural circuits do not work in isolation, and researchers need to further elucidate how complex neural circuits collaborate to control visceral pain. Functional ultrasound imaging may represent an unprecedented opportunity to detect pain and emotional processes in the whole brain in real time while awake and combine these data with in-depth anatomical information on neural circuit mechanisms in animal models^202^.

## Conclusion

In summary, substantial research has been conducted on the neural circuits involved in visceral pain. In this review, we describe the neural circuits that regulate visceral pain to deepen our understanding of visceral pain. With the development of additional high-tech tools and the combined application of various neuroscience technologies, great progress will be made in visceral pain research.

### Reporting summary

Further information on research design is available in the Nature Portfolio Reporting Summary linked to this article.

### Supplementary information

Reporting Summary
